# *N*,*N*′-Bis[(4-methyl­phen­yl)sulfon­yl]adipamide

**DOI:** 10.1107/S1600536811007756

**Published:** 2011-03-05

**Authors:** Vinola Z. Rodrigues, Sabine Foro, B. Thimme Gowda

**Affiliations:** a Department of Chemistry, Mangalore University, Mangalagangotri 574 199, Mangalore, India; b Institute of Materials Science, Darmstadt University of Technology, Petersenstrasse 23, D-64287 Darmstadt, Germany

## Abstract

In the centrosymmetric title compound, C_20_H_24_N_2_O_6_S_2_, the N—H and C=O bonds are *trans* to each other. In the crystal, inter­molecular N—H⋯O(S) hydrogen bonds link the mol­ecules into zigzag chains running along the *b* axis. The O atom involved in the hydrogen bond has a longer S—O bond than the other O atom bonded to S [1.441 (2) versus 1.428 (2) Å].

## Related literature

For our study of the effect of substituents on the structures of sulfonamides, see: Gowda *et al.* (2005 ▶, 2007 ▶); Rodrigues *et al.* (2011 ▶).
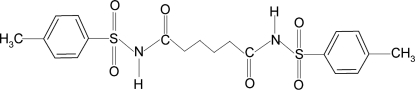


## Experimental

### 

#### Crystal data

C_20_H_24_N_2_O_6_S_2_
*M* *_r_* = 452.53
Triclinic, 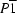

*a* = 6.0011 (9) Å
*b* = 8.765 (1) Å
*c* = 10.144 (2) Å
α = 90.04 (1)°
β = 92.35 (1)°
γ = 98.01 (1)°
*V* = 527.91 (14) Å^3^
*Z* = 1
Mo *K*α radiation
μ = 0.29 mm^−1^
*T* = 293 K
0.48 × 0.12 × 0.09 mm

#### Data collection

Oxford Diffraction Xcalibur diffractometer with a Sapphire CCD detector
Absorption correction: multi-scan (*CrysAlis RED*; Oxford Diffraction, 2009 ▶) *T*_min_ = 0.872, *T*_max_ = 0.974
3355 measured reflections
2122 independent reflections
1651 reflections with *I* > 2σ(*I*)
*R*_int_ = 0.021

#### Refinement

*R*[*F* ^2^ > 2σ(*F* ^2^)] = 0.057
*wR*(*F* ^2^) = 0.141
*S* = 1.08
2122 reflections
140 parameters
1 restraint
H atoms treated by a mixture of independent and constrained refinement
Δρ_max_ = 0.78 e Å^−3^
Δρ_min_ = −0.28 e Å^−3^

### 

Data collection: *CrysAlis CCD* (Oxford Diffraction, 2009 ▶); cell refinement: *CrysAlis RED* (Oxford Diffraction, 2009 ▶); data reduction: *CrysAlis RED*; program(s) used to solve structure: *SHELXS97* (Sheldrick, 2008 ▶); program(s) used to refine structure: *SHELXL97* (Sheldrick, 2008 ▶); molecular graphics: *PLATON* (Spek, 2009 ▶); software used to prepare material for publication: *SHELXL97*.

## Supplementary Material

Crystal structure: contains datablocks I, global. DOI: 10.1107/S1600536811007756/bt5485sup1.cif

Structure factors: contains datablocks I. DOI: 10.1107/S1600536811007756/bt5485Isup2.hkl

Additional supplementary materials:  crystallographic information; 3D view; checkCIF report

## Figures and Tables

**Table 1:** Hydrogen-bond geometry (Å, °)

*D*—H⋯*A*	*D*—H	H⋯*A*	*D*⋯*A*	*D*—H⋯*A*
N1—H1*N*⋯O2^i^	0.84 (2)	2.11 (2)	2.938 (4)	170 (3)

Symmetry code: (i) 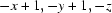.

**Fig. 1.**
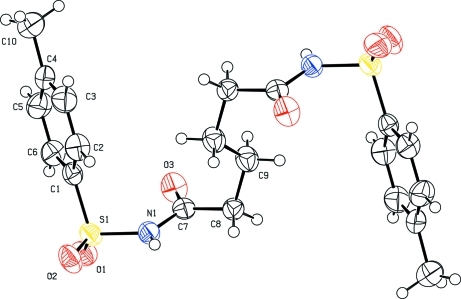
Molecular structure of the title compound, showing the atom labelling scheme and displacement ellipsoids are drawn at the 50% probability level.

**Fig. 2.**
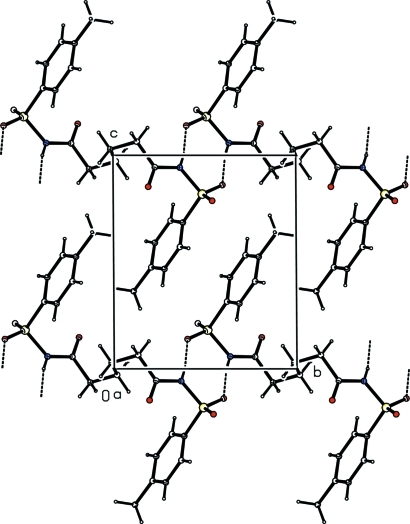
Packing diagram of the title compound with hydrogen bonding shown as dashed lines.

## supplementary crystallographic information

### Comment

The sulfonamide moiety is a constituent of many biologically significant compounds. As a part of studying the effect of substituents on the structures of this class of compounds (Gowda *et al.*, 2005, 2007; Rodrigues *et al.*, 2011), in the present work, the structure of *N*,*N*-bis(4-methylphenylsulfonyl)-adipamide (I) has been determined (Fig.1). The asymmetric unit comprises half of a molecule, the remaining portion being generated *via* an inversion centre, similar to that observed in *N*,*N*-bis(2-methylphenylsulfonyl)-adipamide (II) (Rodrigues *et al.*, 2011). The conformation of the N—H and C=O bonds in the C—SO_2_—NH—C(O)—C—C segment is *anti* to each other and the amide O atom is also *anti* to the H atoms attached to the adjacent C atom. The molecule is bent at the S atom with the C—SO_2_—NH—C(O) torsion angle of -58.5 (3)°, compared to the value of -63.7 (4)° in (II). Further, the S1—N1—C7—C8 and C7—N1—S1—O2 segments are nearly linear. The torsion angles C2—C1—S1—N1 and C6—C1—S1—N1 are, respectively, -60.6 (3)° and 120.3 (3)°. The corresponding values in (II) are -71.3 (4)° and 106.9 (4)°, respectively.

The dihedral angle between the planes of the benzene ring and the SO_2_—NH—C(O)—C—C segment in (I) is 72.0 (1)°, compared to the value of 89.9 (1)° in (II).

N—H···O2(S) H-bond formation results in an S=O2 bond longer than the S=O1 bond. A series of N—H···O(S) intermolecular hydrogen bonds (Table 1) link the molecules into infinite chains running in the *b*-axis direction (Fig. 2).

### Experimental

*N*,*N*-Bis(4-methylphenylsulfonyl)-adipamide was prepared by refluxing a mixture of adipic acid (0.01 mol) with *p*-toluenesulfonamide (0.02 mol) and POCl_3_ for 1 hr on a water bath. The reaction mixture was allowed to cool and added ether to it. The solid product obtained was filtered, washed thoroughly with ether and hot ethanol. The compound was recrystallized to the constant melting point and was characterized by its infrared and NMR spectra.

Needle like colorless single crystals used in the X-ray diffraction studies were grown by a slow evaporation of a solution of the compound in ethanol at room temperature.

### Refinement

The H atom of the NH group was located in a difference map and later restrained to the distance N—H = 0.86 (2) Å. All other H atoms were positioned with idealized geometry using a riding model with aromatic C—H distance = 0.93 Å, methylene C—H = 0.97 Å and methyl C—H = 0.96 Å. All H atoms were refined with isotropic displacement parameters  set to 1.2 times of the *U*_eq_ of the parent atom.

### Crystal data

**Table:**

C_20_H_24_N_2_O_6_S_2_	*Z* = 1
*M**_r_* = 452.53	*F*(000) = 238
Triclinic, *P*1	*D*_x_ = 1.423 Mg m^−^^3^
Hall symbol: -P 1	Mo *K*α radiation, λ = 0.71073 Å
*a* = 6.0011 (9) Å	Cell parameters from 1299 reflections
*b* = 8.765 (1) Å	θ = 3.1–28.0°
*c* = 10.144 (2) Å	µ = 0.29 mm^−^^1^
α = 90.04 (1)°	*T* = 293 K
β = 92.35 (1)°	Needle, colourless
γ = 98.01 (1)°	0.48 × 0.12 × 0.09 mm
*V* = 527.91 (14) Å^3^	

### Data collection

**Table:**

Oxford Diffraction Xcalibur diffractometer with a Sapphire CCD detector	2122 independent reflections
Radiation source: fine-focus sealed tube	1651 reflections with *I* > 2σ(*I*)
graphite	*R*_int_ = 0.021
Rotation method data acquisition using ω scans	θ_max_ = 26.4°, θ_min_ = 3.1°
Absorption correction: multi-scan (*CrysAlis RED*; Oxford Diffraction, 2009)	*h* = −6→7
*T*_min_ = 0.872, *T*_max_ = 0.974	*k* = −10→7
3355 measured reflections	*l* = −12→12

### Refinement

**Table:**

Refinement on *F*^2^	Primary atom site location: structure-invariant direct methods
Least-squares matrix: full	Secondary atom site location: difference Fourier map
*R*[*F*^2^ > 2σ(*F*^2^)] = 0.057	Hydrogen site location: inferred from neighbouring sites
*wR*(*F*^2^) = 0.141	H atoms treated by a mixture of independent and constrained refinement
*S* = 1.08	*w* = 1/[σ^2^(*F*_o_^2^) + (0.0507*P*)^2^ + 0.6262*P*] where *P* = (*F*_o_^2^ + 2*F*_c_^2^)/3
2122 reflections	(Δ/σ)_max_ < 0.001
140 parameters	Δρ_max_ = 0.78 e Å^−^^3^
1 restraint	Δρ_min_ = −0.28 e Å^−^^3^

### Special details

**Table:**

Experimental. CrysAlis RED (Oxford Diffraction, 2009) Empirical absorption correction using spherical harmonics, implemented in SCALE3 ABSPACK scaling algorithm.
Geometry. All e.s.d.'s (except the e.s.d. in the dihedral angle between two l.s. planes) are estimated using the full covariance matrix. The cell e.s.d.'s are taken into account individually in the estimation of e.s.d.'s in distances, angles and torsion angles; correlations between e.s.d.'s in cell parameters are only used when they are defined by crystal symmetry. An approximate (isotropic) treatment of cell e.s.d.'s is used for estimating e.s.d.'s involving l.s. planes.
Refinement. Refinement of *F*^2^ against ALL reflections. The weighted *R*-factor *wR* and goodness of fit *S* are based on *F*^2^, conventional *R*-factors *R* are based on *F*, with *F* set to zero for negative *F*^2^. The threshold expression of *F*^2^ > σ(*F*^2^) is used only for calculating *R*-factors(gt) *etc*. and is not relevant to the choice of reflections for refinement. *R*-factors based on *F*^2^ are statistically about twice as large as those based on *F*, and *R*- factors based on ALL data will be even larger.

### Fractional atomic coordinates and isotropic or equivalent isotropic displacement parameters (Å^2^)

**Table:**

	*x*	*y*	*z*	*U*_iso_*/*U*_eq_	
C1	0.3932 (5)	0.6198 (3)	0.3150 (3)	0.0359 (6)	
C2	0.6161 (5)	0.6864 (4)	0.3058 (4)	0.0474 (8)	
H2	0.6946	0.6720	0.2306	0.057*	
C3	0.7186 (6)	0.7738 (4)	0.4098 (4)	0.0550 (9)	
H3	0.8665	0.8208	0.4031	0.066*	
C4	0.6072 (6)	0.7940 (4)	0.5246 (3)	0.0521 (9)	
C5	0.3872 (6)	0.7224 (4)	0.5321 (3)	0.0550 (9)	
H5	0.3107	0.7327	0.6088	0.066*	
C6	0.2789 (5)	0.6363 (4)	0.4287 (3)	0.0457 (8)	
H6	0.1308	0.5898	0.4353	0.055*	
C7	0.1735 (4)	0.7710 (3)	0.0561 (3)	0.0366 (7)	
C8	0.2257 (5)	0.8691 (3)	−0.0642 (3)	0.0392 (7)	
H8A	0.0870	0.8975	−0.1030	0.047*	
H8B	0.2921	0.8100	−0.1292	0.047*	
C9	0.3879 (5)	1.0149 (4)	−0.0284 (4)	0.0483 (8)	
H9A	0.4117	1.0770	−0.1070	0.058*	
H9B	0.3184	1.0743	0.0350	0.058*	
C10	0.7223 (9)	0.8915 (5)	0.6365 (4)	0.0829 (14)	
H10A	0.7018	0.9971	0.6227	0.099*	
H10B	0.8802	0.8831	0.6400	0.099*	
H10C	0.6582	0.8565	0.7181	0.099*	
N1	0.2657 (5)	0.6347 (3)	0.0575 (3)	0.0411 (6)	
H1N	0.353 (5)	0.614 (4)	0.000 (3)	0.049*	
O1	0.0322 (4)	0.4586 (3)	0.2128 (2)	0.0569 (7)	
O2	0.3994 (5)	0.4024 (3)	0.1391 (2)	0.0585 (7)	
O3	0.0681 (4)	0.8081 (3)	0.1472 (2)	0.0532 (6)	
S1	0.25952 (14)	0.51241 (8)	0.18165 (8)	0.0417 (2)	

### Atomic displacement parameters (Å^2^)

**Table:**

	*U*^11^	*U*^22^	*U*^33^	*U*^12^	*U*^13^	*U*^23^
C1	0.0382 (15)	0.0335 (14)	0.0367 (15)	0.0053 (12)	0.0077 (12)	0.0048 (12)
C2	0.0387 (17)	0.0537 (19)	0.0501 (19)	0.0044 (14)	0.0134 (14)	0.0067 (15)
C3	0.0404 (18)	0.054 (2)	0.066 (2)	−0.0062 (15)	−0.0037 (16)	0.0106 (18)
C4	0.069 (2)	0.0432 (17)	0.0413 (19)	0.0005 (16)	−0.0104 (16)	0.0102 (14)
C5	0.066 (2)	0.063 (2)	0.0354 (18)	0.0029 (18)	0.0114 (16)	0.0021 (16)
C6	0.0414 (17)	0.0514 (18)	0.0431 (18)	−0.0008 (14)	0.0121 (14)	0.0065 (14)
C7	0.0255 (14)	0.0381 (15)	0.0443 (17)	−0.0019 (12)	−0.0001 (12)	0.0011 (13)
C8	0.0376 (16)	0.0394 (15)	0.0398 (17)	0.0035 (12)	−0.0015 (13)	0.0027 (13)
C9	0.0439 (18)	0.0456 (18)	0.054 (2)	0.0022 (14)	0.0027 (15)	0.0095 (15)
C10	0.113 (4)	0.066 (3)	0.059 (3)	−0.015 (3)	−0.024 (2)	0.006 (2)
N1	0.0521 (16)	0.0372 (13)	0.0349 (14)	0.0079 (12)	0.0069 (11)	0.0008 (11)
O1	0.0529 (14)	0.0520 (14)	0.0595 (15)	−0.0161 (11)	0.0062 (11)	0.0040 (11)
O2	0.0910 (19)	0.0375 (12)	0.0521 (15)	0.0215 (12)	0.0213 (13)	0.0053 (10)
O3	0.0475 (13)	0.0578 (14)	0.0580 (15)	0.0152 (11)	0.0218 (11)	0.0072 (12)
S1	0.0516 (5)	0.0318 (4)	0.0411 (4)	0.0018 (3)	0.0091 (3)	0.0013 (3)

### Geometric parameters (Å, °)

**Table:**

C1—C6	1.383 (4)	C7—C8	1.511 (4)
C1—C2	1.389 (4)	C8—C9	1.529 (4)
C1—S1	1.749 (3)	C8—H8A	0.9700
C2—C3	1.376 (5)	C8—H8B	0.9700
C2—H2	0.9300	C9—C9^i^	1.498 (6)
C3—C4	1.390 (5)	C9—H9A	0.9700
C3—H3	0.9300	C9—H9B	0.9700
C4—C5	1.386 (5)	C10—H10A	0.9600
C4—C10	1.505 (5)	C10—H10B	0.9600
C5—C6	1.378 (5)	C10—H10C	0.9600
C5—H5	0.9300	N1—S1	1.653 (3)
C6—H6	0.9300	N1—H1N	0.837 (18)
C7—O3	1.210 (4)	O1—S1	1.428 (2)
C7—N1	1.385 (4)	O2—S1	1.441 (2)
			
C6—C1—C2	120.6 (3)	C7—C8—H8B	109.4
C6—C1—S1	120.3 (2)	C9—C8—H8B	109.4
C2—C1—S1	119.1 (2)	H8A—C8—H8B	108.0
C3—C2—C1	118.8 (3)	C9^i^—C9—C8	114.2 (3)
C3—C2—H2	120.6	C9^i^—C9—H9A	108.7
C1—C2—H2	120.6	C8—C9—H9A	108.7
C2—C3—C4	121.8 (3)	C9^i^—C9—H9B	108.7
C2—C3—H3	119.1	C8—C9—H9B	108.7
C4—C3—H3	119.1	H9A—C9—H9B	107.6
C5—C4—C3	117.9 (3)	C4—C10—H10A	109.5
C5—C4—C10	121.3 (4)	C4—C10—H10B	109.5
C3—C4—C10	120.8 (4)	H10A—C10—H10B	109.5
C6—C5—C4	121.6 (3)	C4—C10—H10C	109.5
C6—C5—H5	119.2	H10A—C10—H10C	109.5
C4—C5—H5	119.2	H10B—C10—H10C	109.5
C5—C6—C1	119.2 (3)	C7—N1—S1	125.4 (2)
C5—C6—H6	120.4	C7—N1—H1N	122 (2)
C1—C6—H6	120.4	S1—N1—H1N	112 (2)
O3—C7—N1	121.5 (3)	O1—S1—O2	118.77 (16)
O3—C7—C8	124.2 (3)	O1—S1—N1	110.32 (15)
N1—C7—C8	114.2 (3)	O2—S1—N1	103.00 (14)
C7—C8—C9	111.2 (3)	O1—S1—C1	109.01 (15)
C7—C8—H8A	109.4	O2—S1—C1	109.75 (15)
C9—C8—H8A	109.4	N1—S1—C1	105.04 (13)
			
C6—C1—C2—C3	−2.4 (5)	C7—C8—C9—C9^i^	61.1 (5)
S1—C1—C2—C3	178.4 (3)	O3—C7—N1—S1	−3.7 (4)
C1—C2—C3—C4	1.7 (5)	C8—C7—N1—S1	173.6 (2)
C2—C3—C4—C5	0.2 (5)	C7—N1—S1—O1	58.8 (3)
C2—C3—C4—C10	−179.4 (3)	C7—N1—S1—O2	−173.4 (3)
C3—C4—C5—C6	−1.3 (5)	C7—N1—S1—C1	−58.5 (3)
C10—C4—C5—C6	178.3 (3)	C6—C1—S1—O1	2.0 (3)
C4—C5—C6—C1	0.5 (5)	C2—C1—S1—O1	−178.8 (2)
C2—C1—C6—C5	1.4 (5)	C6—C1—S1—O2	−129.6 (3)
S1—C1—C6—C5	−179.5 (3)	C2—C1—S1—O2	49.5 (3)
O3—C7—C8—C9	67.3 (4)	C6—C1—S1—N1	120.3 (3)
N1—C7—C8—C9	−109.9 (3)	C2—C1—S1—N1	−60.6 (3)

Symmetry codes: (i) −*x*+1, −*y*+2, −*z*.

### Hydrogen-bond geometry (Å, °)

**Table:**

*D*—H···*A*	*D*—H	H···*A*	*D*···*A*	*D*—H···*A*
N1—H1N···O2^ii^	0.84 (2)	2.11 (2)	2.938 (4)	170 (3)

Symmetry codes: (ii) −*x*+1, −*y*+1, −*z*.
